# Ninjurin 2 overexpression promotes human colorectal cancer cell growth *in vitro* and *in vivo*

**DOI:** 10.18632/aging.102336

**Published:** 2019-10-09

**Authors:** Gang Li, Li-Na Zhou, Han Yang, Xixi He, Yuxia Duan, Fang Wu

**Affiliations:** 1 Department of Chemoradiation Oncology, The First Affiliated Hospital of Wenzhou Medical University, Wenzhou, China; 2 Department of Radiotherapy and Oncology, Affiliated Kunshan Hospital of Jiangsu University, Kunshan, China; 3 Department of Gastroenterology, The First Affiliated Hospital of Wenzhou Medical University, Wenzhou, China; 4 Department of Radiology, The First Affiliated Hospital of Wenzhou Medical University, Wenzhou, China

**Keywords:** colorectal cancer, Ninjurin 2, receptor tyrosine kinases, signalings

## Abstract

Ninjurin 2 (NINJ2) is a novel adhesion molecule. Its expression and potential function in human colorectal cancer (CRC) cells are studied. We show that NINJ2 is overexpressed in established (HT-29) and primary CRC cells and in human colon cancer tissues. Its expression level is low in colon epithelial cells and normal colon tissues. NINJ2 shRNA or knockout (by CRSIPR/Cas9) potently inhibited human CRC cell survival and proliferation, while significantly inducing cell apoptosis. Conversely, lentivirus-mediated NINJ2 overexpression promoted CRC cell proliferation. NINJ2 co-immunoprecipitated with multiple RTKs (EGFR, PDGFRα/β and FGFR) in CRC cells and human colon cancer tissues. In HT-29 cells, RTKs’ downstream signalings, Akt and Erk, were significantly inhibited by NINJ2 shRNA or knockout, but augmented following ectopic NINJ2 overexpression. *In vivo*, NINJ2-silenced or NINJ2-knockout CRC xenografts grew significantly slower than the control xenografts. Akt-Erk activation was largely inhibited in CRC xenografts with NINJ2 silencing or knockout. Taken together, NINJ2 overexpression promotes CRC cell growth *in vitro* and *in vivo*.

## INTRODUCTION

Colorectal cancer (CRC) is a common human malignancy and a major public health problem [1, 2], causing significant human mortalities each year [3, 4]. Over the past decades, several molecularly-targeted agents have been applied for the treatment of CRC patients, yet their efficiencies in patients with advanced metastatic and/or recurrent CRCs are far from satisfactory [1]. The molecule heterogeneity of CRC impedes treatment with specific molecularly-targeted agents [5]. Therefore, it is extremely important to further explore the pathological mechanisms of CRC oncogenesis and progression, and to develop possible novel molecularly-targeted therapies [1].

Ninjurin 2 (NINJ2, also known as nerve injury-induced protein 2) is the homolog of ninjurin1 (NINJ1) [6]. *NINJ2 gene* is located on chromosome 12p13 [6]. NINJ2 and NINJ1 share conserved hydrophobic regions in the transmembrane domain [6]. Studies have proposed that NINJ2 is important for nerve regeneration following nerve injury [6, 7]. NINJ2 is upregulated in Schwann cells surrounding the distal segment of injured nerve, promoting neurite outgrowth [6, 7]. NINJ2 is widely expressed in human tissues, although its expression levels are relatively low in the colon tissues [8]. NINJ2 expression and potential function in CRC and other human cancers have not been studied. The results of the current study show that NINJ2 overexpression promotes CRC cell growth *in vitro* and *in vivo*.

## RESULTS

### NINJ2 upregulation in human CRC cells and tissues

**Figure 1:**
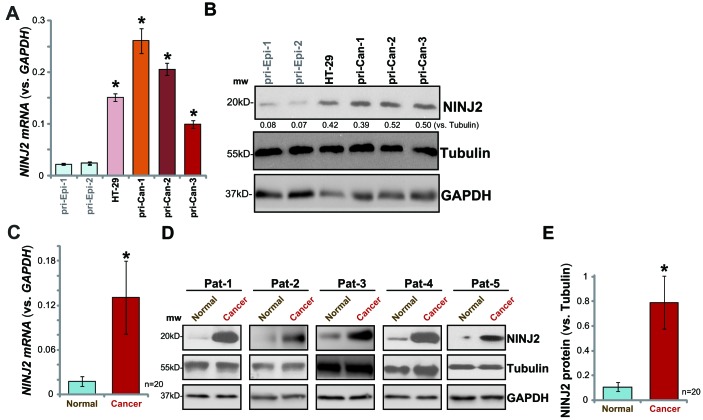
**NINJ2 upregulation in human CRC cells and tissues.** *NINJ2 mRNA* and protein levels in HT-29 cells, primary human colon cancer cells (“pri-Can-1/-2/-3”) and primary human colon epithelial cells (“pri-Epi-1/-2”) were tested by qPCR (**A**) and Western blotting (**B** and **C**), respectively. A total of twenty (20) pairs of human colon cancer tissues (“Cancer”) and paired surrounding normal colon epithelial tissues (“Normal”) were homogenized anddissolved in tissue lysis buffer, *NINJ2 mRNA* and protein expressions were tested by qPCR (**C**) and Western blotting (**D** and **E**), respectively. “Pat” stands for “Patient No.” (**D**). “mw” stands for molecular weight (same for all figures). *NINJ2 mRNA* was normalized to *GAPDHmRNA*. NINJ2 proteinwas normalized to the loading control Tubulin. Bars stand for mean ± SD (same for all Figures).* *P*< 0.05 *vs.*“pri-Epi-1” cells (**A** and **B**) or “Normal” tissues (**C** and **E**).

The current study aims to test the expression and potential function of NINJ2 in CRC cells. qPCR assay was employed to test *NINJ2 mRNA* levels. Results in Figure 1A demonstrated that significant *NINJ2 mRNA* expression was detected in established HT-29 CRC cells. Further, in the primary human colon cancer cells, derived from three different colon cancer patients (“pri-Can-1/-2/-3”), relatively high *NINJ2 mRNA* levels were detected (Figure 1A). On the contrary, *NINJ2 mRNA* levels were low in the primary human colon epithelial cells (“pri-Epi-1/2”, derived from two different donors) (Figure 1A). NINJ2 protein levels were tested by Western blotting assays. In line with the *mRNA* results, NINJ2 protein levels were significantly higher in HT-29 cells and primary colon cancer cells, as compared with its levels in the colon epithelial cells (Figure 1B).

*NINJ2 mRNA* levels in a total of twenty (20) human colon cancer tissues (“Cancer”) and paracancer normal colon epithelial tissues (“Normal”) were analyzed. As shown, *NINJ2 mRNA* levels were significantly upregulated in the colon cancer tissues (Figure 1C). Its levels were low in colon epithelial tissues (Figure 1C). Western blotting analyses confirmed significant NINJ2 protein upregulation in cancer tissues (representative tissues from five independent patients were shown, Figure 1D). Quantitative analyses of blotting results of all twenty pairs of tissues confirmed that NINJ2 protein levels are significantly higher in colon cancer tissues (*P*< 0.05 *vs.* colon epithelial tissues, Figure 1E). Together, these results show that NINJ2 is upregulated in human CRC cells and tissues.

### NINJ2 shRNA inhibits human CRC cell survival and proliferation

**Figure 2:**
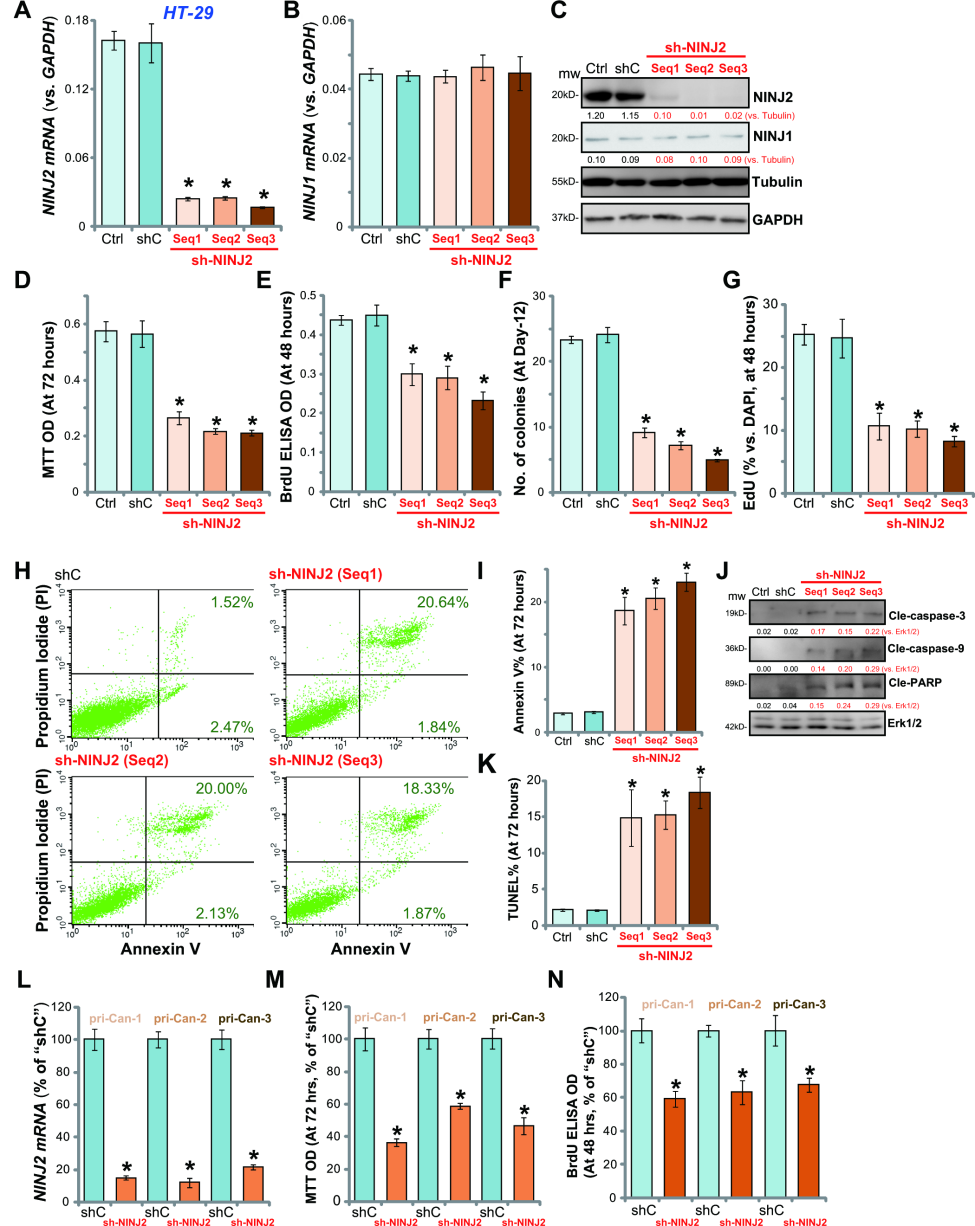
**NINJ2 shRNA inhibits human CRC cell survival and proliferation.** HT-29 cells (**A**–**K**) or the primary human colon cancer cells (“pri-Can-1/-2/-3”, L-N) were infected with lentiviral particles encoding applied NINJ2 shRNA (“Seq1/2/3”) or non-sense control shRNA (“shC”), stable cells were established following puromycin selection; Expression of *NINJ2 mRNA* (**A** and **L**), *NINJ1 mRNA* (**B**) and listed proteins (**C**) were shown; Cell survival was tested by MTT assay (**D** and **M**); Cell proliferation was tested by BrdU incorporation assay (**E** and **N**), soft agar colony formation assay (**F**) and EdU staining (**G**); Cell apoptosis was tested by Annexin V-PI FACS assay (**H**, results quantified in **I**), Western blotting of apoptosis-related proteins (**J**) and TUNEL staining (**K**). For all the *in vitro* functional assays, the exact same number of viable cells with different genetic modifications were initially plated into each well/dish (at Day-0, same for all figures). NINJ1 and NINJ2were normalized to the loading control Tubulin (**C**). “Ctrl” stands for the parental control cells (same for all Figures). For each assay, n=5. * *P*< 0.05 *vs.* “shC” cells. Experiments in this figure were repeated three times, and similar results were obtained. Bar= 200 μm (**G** and **K**).

In order to study the potential effect of NINJ2 on the function of CRC cells, shRNA strategy was utilized. As described, each of the three NINJ2 shRNAs, with non-overlapping sequences (“Seq1/2/3”, listed in Table-1), was individually packed to lentiviral construct, and transfected to HT-29 CRC cells. Following selection by puromycin, the stable cell lines were established, which were named as “sh-NINJ2 (Seq1/2/3)”. By analyzing *NINJ2 mRNA* levels, we show that each of the applied shRNA led to 80–90% reduction of *NINJ2 mRNA* in stable cells (Figure 2A). *NINJ1 mRNA* levels were unchanged by the applied NINJ2 shRNAs (Figure 2B). A significant NINJ2 protein downregulation was detected as well in stable HT-29 cells with NINJ2 shRNA (Figure 2C). NINJ1 protein levels were also unchanged (Figure 2C).

To study the potential effect of NINJ2 knockdown on HT-29 cell functions, MTT viability assay was performed. Results demonstrated that NINJ2 knockdown by targeted shRNAs led to over 50% reduction of HT-29 cell viability (Figure 2D). When analyzing cell proliferation by the BrdU incorporation assay and soft agar colony formation assay, we demonstrated that the applied NINJ2 shRNAs significantly inhibited HT-29 cell proliferation (Figure 2E and 2F). The BrdU ELISA ODs (Figure 2E) and the number of colonies (Figure 2F) were significantly decreased in NINJ2 shRNA-expressing HT-29 cells. Furthermore, EdU incorporation was also significantly suppressed by NINJ2 shRNA (Figure 2G).

Cell apoptosis was tested as well. As demonstrated, each of the applied NINJ2 shRNA (“Seq1/2/3”) increased the percentage of Annexin V-positive HT-29 cells (Figure 2H and 2I). NINJ2 silencing induced cleavages of caspase-3, caspase-9 and PARP in HT-29 cells (Figure 2J). Furthermore, in NINJ2-silenced HT-29 cells, the TUNEL percentage (% *vs.* DAPI) was increased (Figure 2K). These results show that NINJ2 knockdown by targeted shRNA induced viability reduction, proliferation inhibition and apoptosis activation in HT-29 cells. The non-sense scramble control shRNA (“shC”) had no significant effect on NINJ1/NINJ2 expression (Figure 2A–2C) and HT-29 cell function (Figure 2D–2K).

The primary human colon cancer cells (“pri-Can-1/-2/-3”) were infected with the lentivirus with NINJ2 shRNA (“Seq3”). As shown, the NINJ2 shRNA led to dramatic inhibition of *NINJ2 mRNA* in the primary cancer cells (Figure 2L). Consequently, the viability (MTT OD, Figure 2M) and proliferation (BrdU ELISA OD, Figure 2N) were decreased by NINJ2 shRNA in the primary cancer cells. Together, these results show that NINJ2 silencing by targeted shRNA inhibits survival and proliferation of established/primary human CRC cells.

### NINJ2 knockout inhibits HT-29 cell survival and proliferation

**Figure 3:**
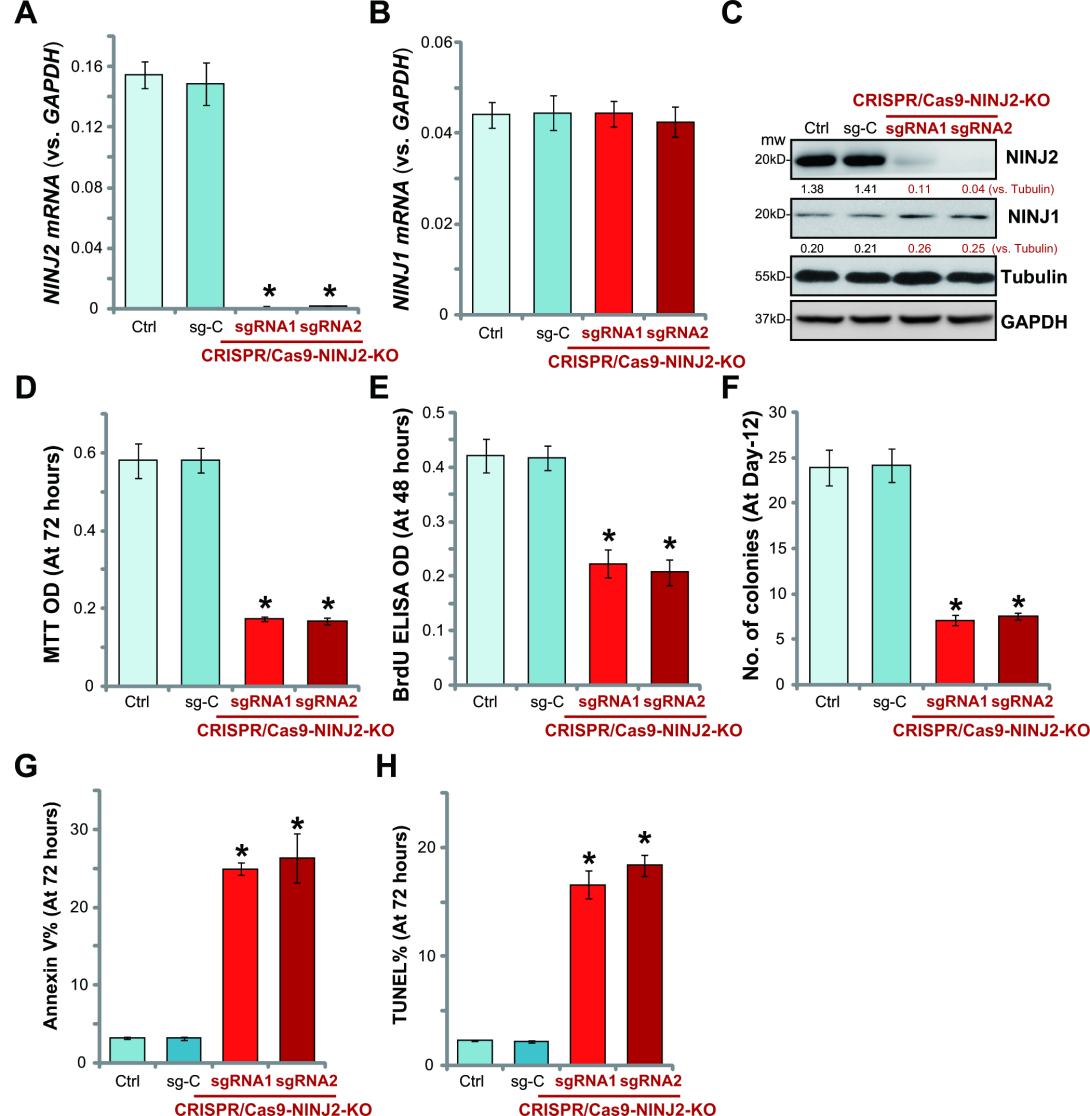
**NINJ2 knockout inhibits HT-29 cell survival and proliferation.** HT-29 cells were transfected with the lenti-CRISPR/Cas9-KO constructs, with non-overlapping sgRNAs against NINJ2 (“sgRNA1/2”) or the non-sense control sgRNA (“sg-C”), stable cells were established via FACS sorting plus puromycin selection; Expression of *NINJ2 mRNA* (**A**), *NINJ1 mRNA* (**B**) and listed proteins (**C**) were shown; Cells were cultured for the indicated time periods, cell survival was tested by MTT assay (**D**); Cell proliferation was tested by BrdU incorporation assay (**E**) and soft agar colony formation assay (**F**); Cell apoptosis was tested by the Annexin V-PI FACS assay (**G**) and TUNEL staining assay (**H**). NINJ1 and NINJ2were normalized to the loading control Tubulin (**C**). For each assay, n=5. * *P*< 0.05 *vs.* “sg-C” cells. Experiments in this figure were repeated three times, and similar results were obtained.

Next, the CRISPR/Cas9 method was applied to knockout NINJ2. Two lenti-CRISPR/Cas9-KO constructs, containing non-overlapping sgRNAs (“sgRNA1/2”) against *NINJ2*, were utilized. Each of the construct was transfected to HT-29 cells. Stable cells were established via FACS GFP sorting plus puromycin selection (see Methods). Analyzing *NINJ2 mRNA* in the stable cells confirmed that the CRISPR/Cas9 NINJ2 KO constructs led to almost complete depletion of *NINJ2 mRNA* in stable cells (Figure 3A). *NINJ1 mRNA* level was unchanged (Figure 3B). Over 95% reduction of NINJ2 protein levels were noticed in the stable HT-29 cells with CRISPR/Cas9 NINJ2 KO constructs (Figure 3C), where NINJ1 protein levels were unchanged (Figure 3C).

Significantly, NINJ2 depletion by the CRISPR/Cas9-KO construct potently inhibited the viability of HT-29 cells, showing significantly decreased MTT ODs (Figure 3D). Furthermore, BrdU ELISA assay and soft agar colony formation assay results demonstrated that proliferation of the NINJ2-knockout cells was inhibited as well (Figure 3E and 3F). Furthermore, the NINJ2-knockout cells showed increased Annexin V percentage (Figure 3G) and TUNEL ratio (Figure 3H), indicating apoptosis activation. Together, these results show that CRISPR/Cas9-mediated NINJ2 knockout inhibited survival and proliferation of HT-29 cells. Notably, the lenti-CRISPR/Cas9 construct with the non-sense control sgRNA (“sg-C”) did not change NINJ1/NINJ2 expression (Figure 3A–3C) and HT-29 cell functions (Figure 3D–3H).

### Ectopic overexpression of NINJ2 promotes CRC cell survival and proliferation

**Figure 4:**
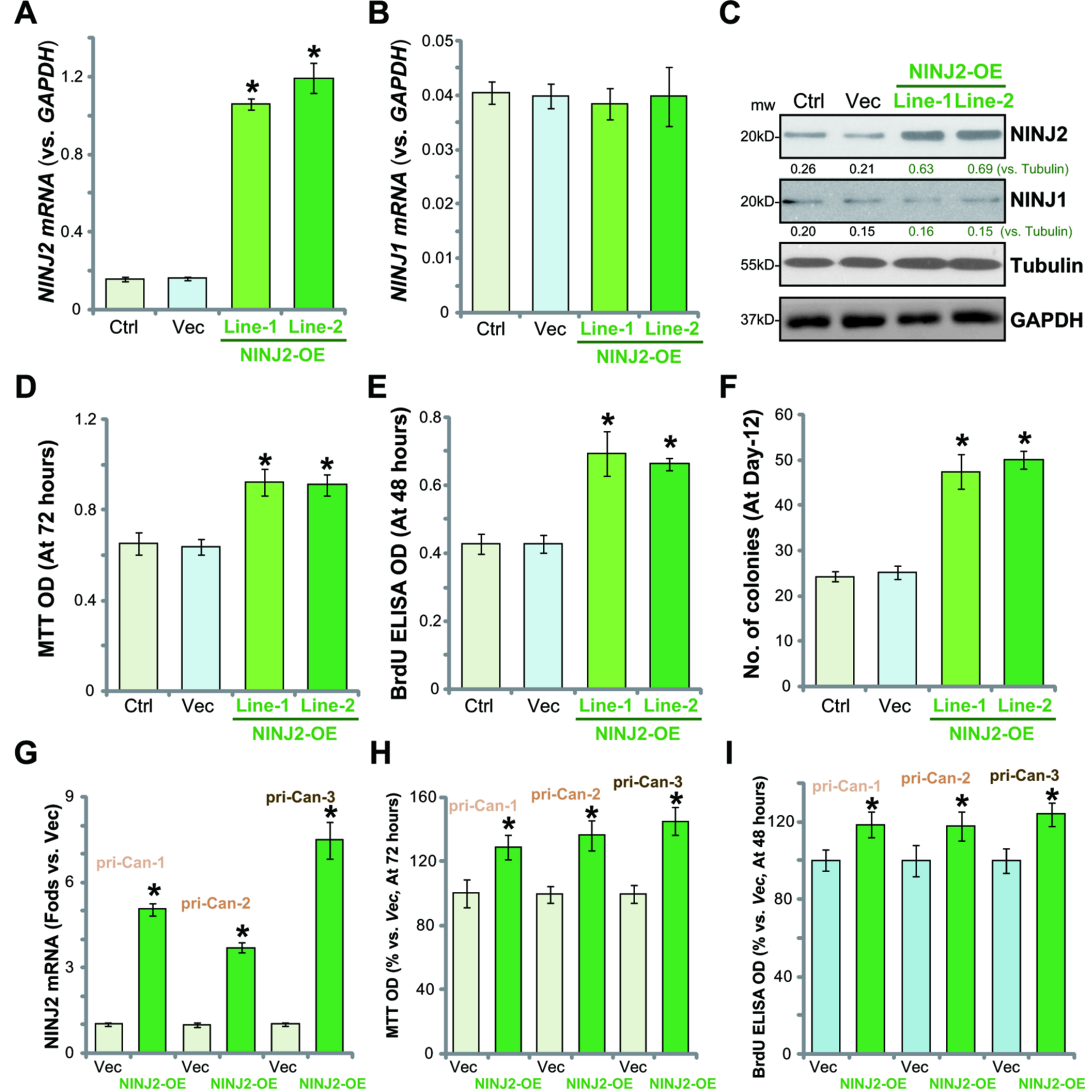
**Ectopic overexpression of NINJ2 promotes CRC cell survival and proliferation.** The lentivirus encoding *NINJ2 cDNA* construct was added to HT-29 cells. Via selection by puromycin two stable cell lines (“Line-1/-2”) were established. Control cells were infected with virus encoding empty vector (“Vec”); Expression of *NINJ2 mRNA* (**A**), *NINJ1 mRNA* (**B**) and listed proteins (**C**) were shown; Cells were further cultured for the indicated time periods, cell survival was tested by MTT assay (**D**); Cell proliferation was tested by BrdU incorporation assay (**E**) and soft agar colony formation assay (**F**); The primary human colon cancer cells (“pri-Can-1/-2/-3”) were infected with lentivirus encoding *NINJ2 cDNA* construct (“NINJ2-OE”) or the empty vector (“Vec”); Cells were cultured for the indicated time periods; *NINJ2 mRNA* expression was tested by qPCR assay (**G**); Cell survival and proliferation were tested by the MTT assay (**H**) and BrdU incorporation assay (**I**), respectively. For each assay, n=5. NINJ1 and NINJ2were normalized to the loading control Tubulin (**C**). * *P*< 0.05 *vs.*“Vec” cells. Experiments in this figure were repeated three times, and similar results were obtained.

Based on the above results, we hypothesized that forced-overexpression of NINJ2 could possibly promote CRC cell progression. To test this hypothesis, the lentivirus encoding *NINJ2 cDNA* was added to HT-29 cells. Via puromycin selection two stable cell lines (“Line-1/-2”) were established. qPCR assay results confirmed that *NINJ2 mRNA* levels increased over 10 times in the NINJ2-overexpressed (“NINJ2-OE”) cells (Figure 4A), where *NINJ1 mRNA* was unchanged (Figure 4B). NINJ2 protein levels were also significantly increased in the two stable lines of NINJ2-OE cells (Figure 4C).

As compared to the vector control cells (“Vec”), NINJ2-OE HT-29 cells showed significantly increased viability (MTT OD, Figure 4D), BrdU incorporation (Figure 4E) and colony formation (Figure 4F). Similar results were obtained in the primary human colon cancer cells (“pri-Can-1/-2/-3”), where lentivirus-mediated overexpression of NINJ2 (“NINJ2-OE”, Figure 4G) increased MTT OD (Figure 4H) and BrdU incorporation (Figure 4I). These results confirmed that ectopic NINJ2 overexpression promoted CRC cell survival and proliferation.

To further support the hypothesis, the rescue experiments were performed. CRISPR/Cas9-KO HT-29 cells (by sgRNA1, see Figure 3) were further infected with the lentivirus encoding *NINJ2 cDNA.* After selection through the puromycin-containing medium two stable cell lines (“NINJ2-OE-Line-1/-2”) were established. As shown,*NINJ2 mRNA* (Supplementary Figure 1A) and protein (Supplementary Figure 1B) levels were restored in the stable cells. Significantly, NINJ2 KO-induced proliferation inhibition (BrdU ELISA OD reduction, Supplementary Figure 1C) and apoptosis activation (TUNEL staining increase, Supplementary Figure 1D) were reversed with NINJ2 rescue in HT-29 cells. These results further confirmed the requirement of NINJ2 in promoting HT-29 cell progression.

### NINJ2 forms a complex with multiple receptor tyrosine kinases (RTKs) in CRC cells and colon cancer tissues

**Figure 5:**
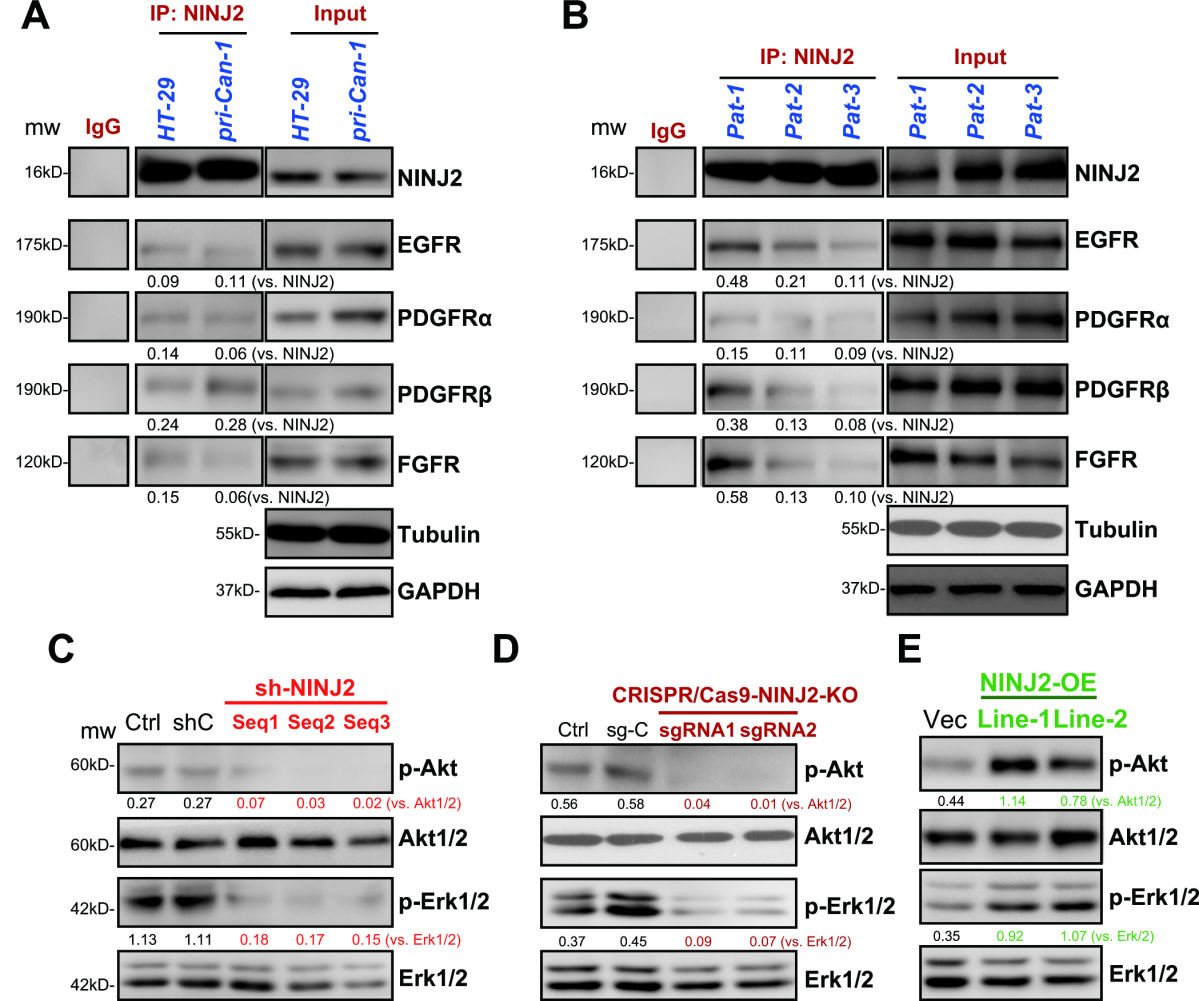
**NINJ2 forms a complex with multiple receptor tyrosine kinases (RTKs) in CRC cells and colon cancer tissues.** In HT-29 cells and primary human colon cancer cells (“pri-Can-1”) the associations between NINJ2 with multiple RTKs (EGFR, PDGFRα, PDGFRβ and FGFR) were tested by co-immunoprecipitation (Co-IP) assays (**A**); “Input” shows expression of RTKs and NINJ2 in total cell lysates (**A**). Fresh human colon tissue lysates from patient-1/-2/-3/ (“Pat-1/2/3”) were subjected to the same Co-IP assay of NINJ2-RTKs associations (**B**), “Input” shows expression of RTKs and NINJ2 in lysates (**B**). Expression of the listed proteins in stable HT-29 cells with applied NINJ2 shRNA (“Seq1/2/3”) or non-sense control shRNA (“shC”) were shown (**C**). Stable HT-29 cells with the lenti-CRISPR/Cas9-KO constructs (with NINJ2 sgRNA1/2, (**D**) or NINJ2 cDNA construct (“NINJ2-OE”, two lines, **E**) were subjected to the same Western blotting assay of listed proteins. NINJ2-bound RTKs (EGFR, PDGFRα, PDGFRβ and FGFR) were quantified (**A** and **B**). Akt and Erk phosphorylations were normalized to total proteins (**C**–**E**). Experiments in this figure were repeated three times, and similar results were obtained.

Simultaneous activation of multiple RTKs will induce persistent activation of downstream PI3K-Akt-mTOR and Erk-MAPK signalings, leading to CRC oncogenesis and progression [9, 10]. Several of these RTKs, including EGFR (epidermal growth factor receptor) [11], PDGFRα(platelet-derived growth factor receptor α), PDGFRβ and FGFR (fibroblast growth factor receptor), are important oncogenes of CRC [9, 10]. NINJ2 is a novel adhesion molecule located on the cell surface [6, 7]. To test if there is a possible interaction between NINJ2 and cell surface RTKs in CRC cells, co-immunoprecipitation (co-IP) assay was performed. As shown, in both HT-29 cells and primary human colon cancer cells (“pri-Can-1”), NINJ2 co-immunoprecipitated with EGFR, PDGFRα, PDGFRβ and FGFR (Figure 5A). NINJ2 associations with these RTKs were also detected in fresh human colon cancer tissue lysates (Figure 5B). Thus, NINJ2 could possibly form a complex with multiple RTKs in CRC cells and human colon cancer tissues.

To explore whether NINJ2 is important for the function of RTKs, we tested RTKs’ downstream signalings, Akt and Erk1/2. As shown, NINJ2 knockdown by the three shRNAs (see Figure 2) significantly inhibited phosphorylations of Akt (at Ser-473) and Erk1/2 in HT-29 cells (Figure 5C). Furthermore, in HT-29 cells, CRISPR/Cas9-mediated NINJ2 knockout (see Figure 3) potently inhibited Akt and Erk1/2 phosphorylations as well (Figure 5D). On the contrary, Akt and Erk1/2 phosphorylations were augmented in the two lines of NINJ2-OE HT-29 cells (Figure 5E). These results indicate that NINJ2, associating with multiple RTKs, is essential for the activation of Akt and Erk1/2 in CRC cells.

### NINJ2 silencing or depletion inhibits HT-29 xenograft growth in SCID mice

**Figure 6:**
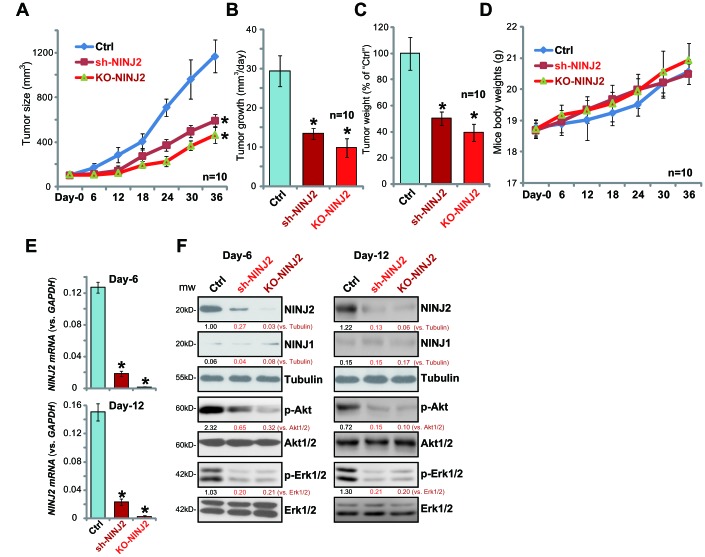
**NINJ2 silencing or depletion inhibits HT-29 xenograft growth in SCID mice.** Stable HT-29 cells (6×10^6^ cells per mouse) with NINJ2 shRNA (“Seq3”) or lenti-CRISPR/Cas9-NINJ2 KO construct (“sgRNA-2”), as well as the parental control HT-29 cells (“Ctrl”), were *s.c.* inoculated into the flanks of the SCID mice. When each tumor was around 100 mm^3^ in volume, the recording was started. Tumor volumes (**A**) and mice body weights (**D**) were recorded every 6 days for a total of 36 days; Estimated daily tumor growth (in mm^3^ per day) was calculated (**B**); At Day-36, each tumor was isolated and weighted individually (**C**); At Day-6 and Day-12, one tumor of each group was separated. The total six tumors were homogenized anddissolved in the tissue lysis buffer, *NINJ2 mRNA* and listed proteins were tested by by qPCR assay (**E**) and Western blotting assay (**F**). Expression of the listed proteins were quantified, normalizing to the loading control protein (**F**). For each group, n=10. *P<0.05 *vs.*“Ctrl” tumors.

The potential effect of NINJ2 on CRC cell progression *in vivo* was tested. Using a previously-described xenograft SCID mouse model [12], NINJ2 shRNA (“Seq3”)-expressing HT-29 cells (“sh-NINJ2”), NINJ2-knockout HT-29 cells (“KO-NINJ2”, using “sgRNA2”) as well as the parental control HT-29 cells (“Ctrl”) were *s.c.* inoculated to the flanks of the SCID mice. Tumor xenografts were established within three weeks, when the volume of each tumor was close to 100 mm^3^ (marked as “Day-0”). Tumor recordings then started every six days for a total of 36 days. Tumor growth curve results demonstrated that sh-NINJ2 HT-29 xenografts and the KO-NINJ2 HT-29 xenografts grew significantly slower than the control HT-29 xenografts (Figure 6A). The estimated daily tumor growth was also calculated, using the following formula: (Tumor volume at Day-36 subtracting Tumor volume at Day-36)/36 (days). Results show that NINJ2 shRNA or NINJ2 knockout dramatically inhibited daily growth of HT-29 xenografts (Figure 6B). At day-36, tumors of all three groups were isolated and weighted individually. We show that sh-NINJ2- or KO-NINJ2-HT-29 xenografts weighted significantly lower than the control tumors (Figure 6C). Mice body weight was not significantly different from the three groups (Figure 6D). These results show that NINJ2 silencing or depletion inhibited HT-29 xenograft growth in SCID mice.

To analyzing signaling changes, at recording “Day-6” and “Day-12”, one tumor of each group was separated. The total six tumors were homogenized in the tissue lysis buffer. qPCR assay results confirmed that, as compared to the control tumors, *NINJ2 mRNA* levels were significantly downregulated in sh-NINJ2 HT-29 xenografts (Figure 6E), and its levels were further reduced in the KO-NINJ2 xenografts (Figure 6E). NINJ2 protein levels were also significantly downregulated in sh-NINJ2- and KO-NINJ2-xenografts (as compared to control tumors, Figure 6F). Importantly, p-Akt and p-Erk1/2 levels were also largely inhibited in sh-NINJ2- and KO-NINJ2-tumor tissues (Figure 6F). Therefore, in line with the *in vitro* findings, NINJ2 silencing or depletion inhibited Akt-Erk activation in HT-29 xenograft tissues.

### NINJ2 knockout inhibits primary human colon cancer cell growth *in vivo*

**Figure 7:**
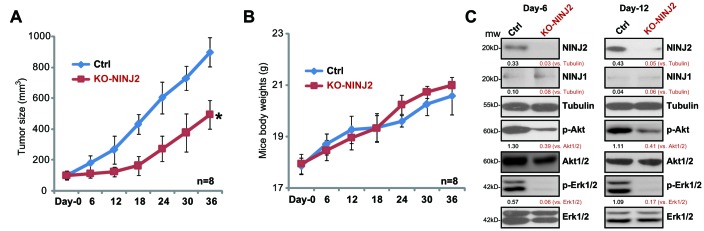
**NINJ2 knockout inhibits primary human colon cancer cell growth *in vivo*.** The stable primary human colon cancer cells (“pri-Can-1”) with lenti-CRISPR/Cas9-NINJ2 KO construct (“KO-NINJ2”, with “sgRNA-2”), or the parental control cells (“Ctrl”), were *s.c.* inoculated into the flanks of the SCID mice (6 × 10^6^ cells per mouse). When each tumor was close 100 mm^3^ in volume, the recording was started (labeled as “Day-0”). Tumor volumes (**A**) and mice body weights (**B**) were recorded every 6 days for a total of 36 days; At recording “Day-6” and “Day-12”, one tumor of each group was separated, tumors were subjected to Western blotting assay of listed proteins (**C**). NINJ1/2 protein expression and Akt-Erk1/2 phosphorylations were quantified (**C**). For each group, n=8. *P<0.05 *vs.*“Ctrl” tumors.

To further support of the activity of NINJ2 in CRC cell progression, the lenti-CRISPR/Cas9-NINJ2 KO construct (with “sgRNA2”, see Figure 3) was transfected to the primary human colon cancer cells (“pri-Can-1”). Stable cells were established via FACS GFP sorting plus puromycin selection. The parental control primary cancer cells and the NINJ2-knockout primary cancer cells were *s.c.* injected to the flanks of SCID mice. The xenografted tumors were established within four weeks (labeled as “Day-0”). By measuring tumor volumes, we show that the xenografts-derived from NINJ2 knockout primary cancer cells (“KO-NINJ2”) grew significantly slower than the xenografts of control cancer cells (“Ctrl”) (Figure 7A). There is no apparent toxicities (fever, sudden weight loss, vomiting *etc*) in the experimental mice, and the body weight was not significantly different among the two groups (Figure 7B).

We next tested NINJ2 expression in the xenograft tissues. At recording “Day-6” and “Day-12”, we isolated one tumor from each group. Western blotting assay results showed that NINJ2 protein levels were significantly downregulated in KO-NINJ2-tumors (Figure 7C). Further, as compared to “Ctrl” tumors, p-Akt and p-Erk1/2 levels were significantly decreased in the KO-NINJ2-tumor lysates (Figure 7C). IHC staining of Day-6 tumors, in Figure 7D, further confirmed Akt inhibition in KO-NINJ2-tumors. These results show that NINJ2 knockout inhibits primary human colon cancer cell growth *in vivo*.

## DISCUSSION

The knowledge of NINJ2 function is very limited [13–15]. The mechanisms underlying NINJ2-mediated functions are also largely unknown. Sporadic studies show that NINJ2 is a cell surface adhesion molecule, which is upregulated in Schwann cells in the distal nerve segment after peripheral nerve injury [6]. NINJ2 can promote neurite outgrowth from dorsal root ganglion neurons via NINJ2-mediated hemophilic cellular interaction [6]. A very recent study by Wang et al., demonstrated that NINJ2 has a pro-inflammatory function in vascular endothelial cells. NINJ2 is shown to directly interact with Toll like receptor 4 (TLR4) to mediate downstream NF-κB (nuclear factor-kappa B) and c-Jun pathway activation [7]. Liu et al.*,* have shown that NINJ2 expression is important for the survival of neuronal cells [16]. Very recently, Jing et al., demonstrated that ectopic NINJ2 overexpression protected neuronal cells from hydrogen peroxide (H_2_O_2_) [17]. To our best knowledge, NINJ2’s expression and potential functions in CRC and other human cancers have not been studied thus far.

The results of the current study indicate that NINJ2 could be a novel and important oncogenic protein in human CRC. First, NINJ2 was significantly upregulated in established and primary human CRC cells (*vs.* normal colon epithelial cells). Furthermore, *NINJ2 mRNA* and protein levels are high in human colon cancer tissues, but were extremely low in normal paracancer colon epithelial tissues. Second, shRNA-mediated knockdown or CRSIPR/Cas9-mediated knockout of NINJ2 significantly inhibited human CRC cell survival and proliferation, while inducing significant cell apoptosis. Third, lentivirus-mediated overexpression of NINJ2 promoted CRC cell growth. Fourth, NINJ2 can associate with multiple oncogenic RTKs, which is essential for the activation of downstream Akt and Erk signalings in CRC cells. Finally, NINJ2-silenced or NINJ2-knockout HT-29 xenografts grew significantly slower than the control tumors. Likewise, NINJ2 knockout inhibited primary human colon cancer cell growth in SCID mice. This evidence clearly indicates that NINJ2 could be a novel and key oncogenic protein of CRC.

In human CRC cells, simultaneous and sustained activation of several RTKs (*i.e.* EGFR, PDGFRα, PDGFRβ and FGFR) shall induce persistent activation of downstream cascades, including PI3K-Akt and Erk-MAPK signalings, which work in a coordinated fashion to promote cancer cell growth, survival, and angiogenesis as well as invasion/migration and apoptosis/death resistances [18]. The anti-CRC agents that target one single RTK often have very limited successes in clinical practices. Regorafenib (BAY 73-4506), a small-molecule multi-kinase inhibitor, has been approved by Food and Drug Administration (FDA) for the treatment of metastatic CRC (mCRC) [18]. Regorafenib is active against several RTKs, showing acceptable efficiency in suppressing human CRC cells [18]. However, the clinical usages of this compound are often associated with significant toxicities even administered at the approved doses [18]. It is therefore an urgent need to identify novel and key proteins in the RTK signaling, which could be novel biomarkers and therapeutic targets.

**Figure 8:**
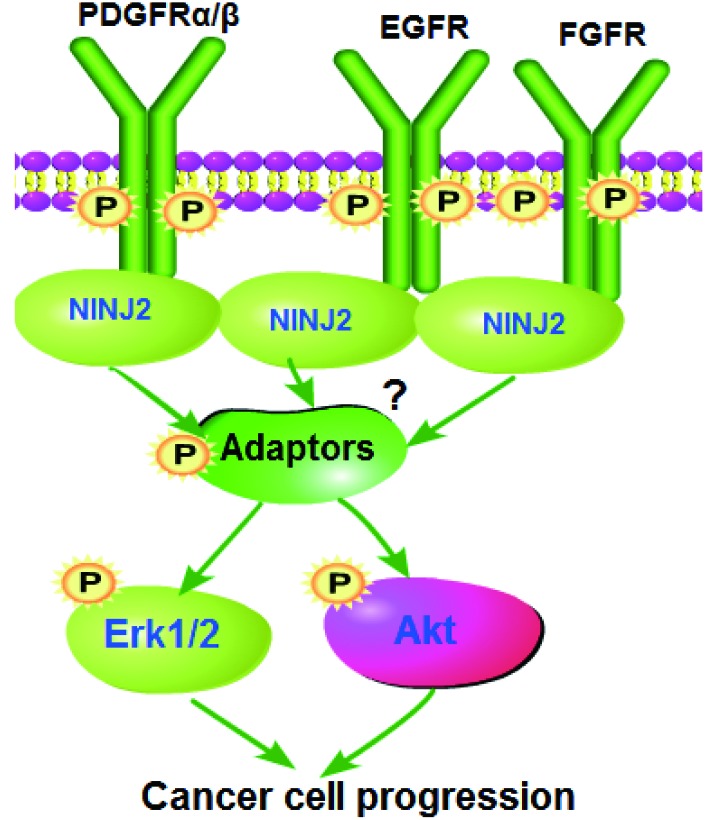
**The proposed signaling pathway of the study.**

This current study implied an essential function of NINJ2 in mediating Akt and Erk activations by RTKs. NINJ2 co-immunoprecipitated with multiple RTKs (EGFR, PDGFRα/β and FGFR) in CRC cells and human colon cancer tissues. Importantly, downstream Akt and Erk activations were significantly inhibited by NINJ2 silencing or knockout, but augmented with ectopic NINJ2 overexpression. Therefore, NINJ2 could be a novel and key adaptor protein for multiple RTKs in CRC cells, mediating downstream Akt and Erk signaling activation (see proposed signaling pathway carton in Figure 8). Targeting NINJ2 can result in better efficiency in blocking downstream signaling activation, thus causing potent inhibition on CRC cells. The underlying signaling mechanisms, however, may warrant further characterizations.

Together, our results show that NINJ2 overexpression promotes CRC cell growth *in vitro* and *in vivo*. NINJ2 could be a novel oncogenic protein and therapeutic target for human CRC.

## MATERIALS AND METHODS

### Chemicals and reagents

Puromycin, polybrene and MTT (3-(4,5-Dimethylthiazol-2-yl)-2,5-diphenyltetrazolium bromide) dye were purchased from Sigma-Aldrich (St. Louis, MO). Fetal bovine serum (FBS) and other cell culture reagents were provided by Hyclone (Logan, UT). Anti-NINJ2 antibody (ab172627) and anti-NINJ1 antibody (ab201647) were obtained from Abcam (Cambridge, MA). All other antibodies were provided by Cell Signaling Tech (Danvers, MA). Lipofectamine 2000, Annexin V and propidium iodide (PI) were purchased from Invitrogen Life Techniques (Carlsbad, CA).

### Cell culture

Culture of HT-29 cells was reported early [12]. The primary human colon cancer cells, derived from three primary colon cancer patients (“pri-Can-1/-2/-3”), as well as the primary human colon epithelial cells (“pri-Epi-1/-2”, from two independent donors) were provided by Dr. Lu [19–21]. The primary human cells were cultured in medium for primary human cells (DMEM, 15% FBS, 10 mg/mL transferrin, 2 mM glutamine, 1 mM pyruvate, 10 mM HEPES, 100 units/mL penicillin/streptomycin, 0.1 mg/mL gentamicin, 0.2 units/mL insulin, 0.1 mg/mL hydrocortisone, and 2 g/L fungizone) [19]. The protocols of using human cells and tissues were according to the principles of Declaration of Helsinki, with approval from the Ethics Board of Wenzhou Medical University (2015-0116). Written informed-consent was obtained from each donor.

**Human tissues**

Twenty (20) primary colon cancer patients(summarized in our previous study [12]) were enrolled. The cancer tissues and the matched surrounding (“paracancer”) normal colon epithelial tissues were separated by the operating microscopes. Fresh tissue specimens were washed, minced, and homogenized by the tissue lysis buffer (Beyotime Biotechnology, Wuxi, China), before further biochemical analyses.

### qPCR assay

Total RNAs of cultured cells or human tissues were extracted by the TRIzol reagents (Sigma). For each assay, 500 ng RNAs were mixed with SYBR Master Mix (Applied Biosystem) and primers (100 nM). The ABI Prism 7900 Fast Real-Time PCR system was utilized for quantitative real-time PCR (qPCR) assays. qPCR quantification was through 2^—ΔCt^ method via the formula: 2^—(Ct of target gene—Ct of reference gene)^, with *GAPDH/ Tubulin mRNA* as the internal control. *mRNA primers* were listed in Table 1.

**Table 1:** Sequences utilized in the study.

**Gene names**	**Sequences**
qPCR mRNA primers	
*GAPDH* Forward	5′-GTCGTGTGAACGGATTTG-3′
*GAPDH* Reverse	5′-AAGATGGTGATGGGCTTCC-3′
*NINJ1* Forward	5′-TCATCTCCATCTCCCTTGTGCT-3′
*NINJ1* Reverse	5′-AGTCCAGCTTGGCGTGCTT-3′
*NINJ2* Forward	5′-CATCCTCTCACTACTACACCACC-3′
*NINJ2* Reverse	5′-CTGGTTGAGTCGCCACTGCTTT-3′
	
NINJ2 shRNA	
Seq1	5′-GGAGCCTGGAGGAGCCCACGCAG-3′
Seq2	5′-TTGAGGGCAGCGAGATGGAATCA-3′
Seq3	5′-CCCATCAACCTGAACCATTACGC-3′
	
	
NINJ2 cDNA	5′-ATGGCAGGTCTGTCCCGCCAGCTGTGTGCTCTCTCCCACCCGAAGAAAGCA GCAGAGACTCAGACGGCGGAGCCTGGAGGAGCCCACGCAGTCTGTTCCCGGC ACCCGGTGCGTGTGAAGGGACTTGAGGGCAGCGAGATGGAATCAGCAAGAG AAAACATCGACCTTCAACCTGGAAGCTCCGACCCCAGGAGCCAGCCCATCAA CCTGAACCATTACGCCACCAAGAAGAGCGTGGCGGAGAGCATGCTGGACGTG GCCCTGTTCATGTCCAACGCCATGCGGCTGAAGGCGGTGCTGGAGCAGGGAC CATCCTCTCACTACTACACCACCCTGGTCACCCTCATCAGCCTCTCTCTGCTCC TGCAGGTGGTCATCGGTGTCCTGCTCGTGGTCATTGCACGGCTGAACCTGAAT GAGGTAGAAAAGCAGTGGCGACTCAACCAGCTCAACAACGCAGCCACCATCT TGGTCTTCTTCACTGTGGTCATCAATGTTTTCATTACAGCCTTCGGGGCACATA AAACAGGGTTCCTGGCTGCCAGGGCCTCAAGGAATCCTCTC-3′
sgRNA sequence	
*NINJ2* Targeted DNA sequence, sgRNA-1	5′-GCATGGCGTTGGACATGAAC-3′
*NINJ2* Targeted DNA sequence, sgRNA-2	5′-TCTTGGTGGCGTAATGGTTC-3′

### Western blotting

Western blotting assay was performed as previously described [22]. For all the assays, the exact same set of lysates (30 μg protein lysates from each treatment in each lane) were run in sister gels to test different proteins. ImageJ software (NIH) was utilized for the quantification of the total gray of each band.

### Co-immunoprecipitation (Co-IP)

For each treatment, a total of 1000 μg protein lysates in 1 mL lysis buffer were pre-cleared by adding IgA/G beads (30 μL, Sigma). Endogenous NINJ2 was precipitated with anti-NINJ2 antibody plus protein IgA/G beads (IP). The NINJ2-bound proteins were subjected to Western blotting analysis.

### Cell viability assay

Routine MTT assays were performed to test the cell viability. Five thousand viable cells per well were initially seeded onto the 96-well tissue culture plates. Following treatment, the MTT optical densities (ODs) at 550 nm were recorded.

### BrdU incorporation assay

As described [12], following the applied treatment, the BrdU incorporation was tested by a BrdU ELISA kit (Roche Diagnostics, #11647229001, Basel, Switzerland) according to the manufacturer’s protocol. The BrdU ELISA absorbance at 405 nm was recorded.

### EdU staining

The detailed protocols for the EdU staining assay were described previously [23, 24]. EdU percentages (EdU*vs.* DAPI, %) of 200 cells per treatment in five random views (under 1: 200 magnification) were recorded.

### Soft agar colony formation

Ten thousand HT-29 cells of different genetic modifications were seeded on the top layer of 0.5% solidified agar (Sigma) in FBS-containing complete medium in 10-cm culture dishes (with the bottom layer containing 1% agar). The complete medium was renewed every two days for a total of 12 days. The number of colonies was counted.

### Annexin V FACS

Briefly, cells were seeded onto the six-well tissue culture plates (3 × 10 ^5^cells per well). Following treatment, cells were harvested, washed, and incubated with Annexin V and PI, each at 5 μg/mL, for 15 min under the dark. Afterwards, cells were analyzed by fluorescent-activated cell sorting (FACS) on a FACSCalibur machine (BD Biosciences).

### TUNEL staining

As described [25], cells were seeded onto the six-well tissue culture plates (3 × 10 ^5^cells per well). A TUNEL (Terminal deoxynucleotidyl transferase dUTP nick end labeling) *In Situ* Cell Death Detection Kit (Roche Diagnostics, Basel, Switzerland), based on labeling of DNA strand breaks, was employed to quantify cell apoptosis. TUNEL percentages (TUNEL *vs.* DAPI, %) of 200 cells per treatment in five random views (under 1: 200 magnification) were recorded.

### NINJ2 short hairpin RNA (shRNA)

The human NINJ2 short hairpin RNA (shRNA) sequence (three non-overlapping NINJ2 shRNA sequences were utilized, listed in Table 1) was inserted into the lenti-pLKO1-puro-GFP vector (Genepharm, Shanghai, China). The construct and lentivirus packaging plasmids were co-transfected to HEK-293 cells by Lipofectamine 2000 to generate lentiviral particles. After filtration and enrichment, the viral particles were added to CRC cells with polybrene. When necessary, the infected cells were cultured in the selection medium with puromycin (5 μg/mL) for five-six more passages (12-15 days). Control cells were infected with the lentiviral particles with scramble control shRNA (Santa Cruz Biotech). Expression of NINJ2 was tested by qPCR assay and Western blotting assay.

### CRISPR/Cas9-mediated NINJ2knockout

The small guide RNA (sgRNA) targeting human *NINJ2* (two different sequences were utilized, listed in Table 1) was inserted into the lenti-CRISPR-GFP-puro plasmid [12]. The construct was then transfected to CRC cells by Lipofectamine 2000. FACS was utilized to sort the GFP-positive cells. The resulting cells were further cultured in the selection medium with puromycin (5 μg/mL) for six passages (12-15 days). NINJ2 knockout in the stable CRC cells was verified by qPCR assay and Western blotting assay. Control cells were transfected with lenti-CRISPR-GFP-puro plasmid with scramble nonsense sgRNA. DNA sequencing was always performed.

### Ectopic NIN2 overexpression

*NINJ2 cDNA* (the sequence was listed in Table-1), synthesized by Genechem (Shanghai, China), was inserted into the hU6-MCS-Ubiquitin-EGFP-IRES-puromycin vector (GV428 [12]) to generate NINJ2 expression vector. The construct and the lentivirus packaging plasmids (Genepharm) were co-transfected to HEK-293 cells to generate viral particles. After filtration and enrichment (10^8^ TU/mL), viral particles were added to CRC cells. The infected cells were cultured in the selection medium with puromycin (5 μg/mL) for five-six more passages (12-15 days). NINJ2 overexpression was verified by qPCR assay and Western blotting assay. Control cells were infected with virus with empty vector [12].

### Xenograft assay

As previously described [12], the female severe combined immunodeficient (SCID) mice (4-5 week-age, 17.5-18.5 g) were provided by the Experimental Animal Center of Wenzhou Medical University (Wenzhou, China). The animals were kept under standard conditions [12]. HT-29 cells or the primary human colon cancer cells, with/without applied genetic modifications, were subcutaneously (*s.c.*) injected into the right flanks of SCID mice (six millions cells each mouse, mixed in 200 μl of Matrigel gel, no FBS). Tumor volume was calculated using the following formula: volume = 0.5328 ×Length × Width × Height (mm^3^). All animal procedures were approved by IACUC of Wenzhou Medical University.

### Statistical analysis

Descriptive statistics including mean and standard deviation (SD) along with one-way ANOVAs were applied to determine significant differences. A 2-tailed paired T test (Excel 2007) was applied to test significance between two treatment groups. *P* values < 0.05 were considered statistically significant.

## Supplementary Material

Supplementary Figure
