# Supplementary material for: Ninjurin 2 overexpression promotes human colorectal cancer cell growth in vitro and in vivo
Source: Aging (Albany NY). 2019 Oct 9;11(19):8526–41. doi: 10.18632/aging.102336 (PMC6814613; doi:10.18632/aging.102336)
Supplement: Supplementary Figure [file aging-11-102336-s001.pdf]

SUPPLEMENTARY FIGURE

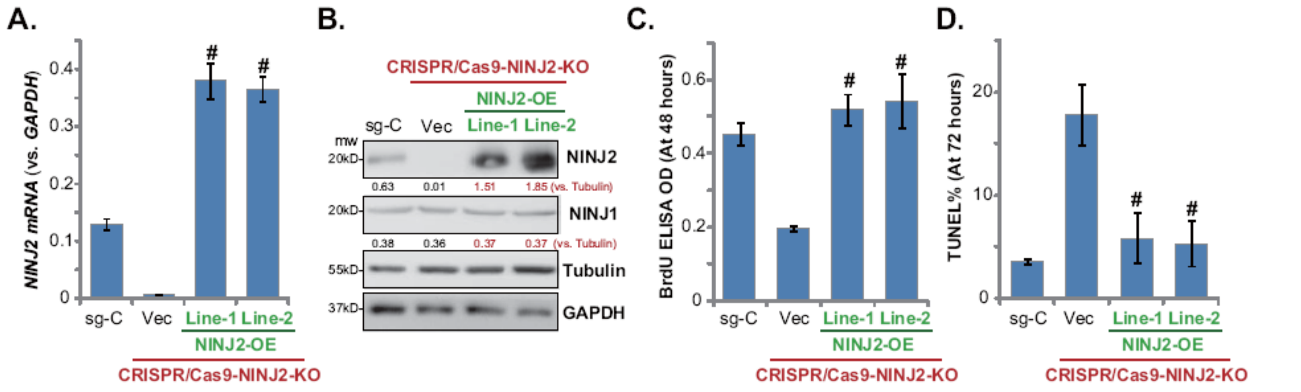

Supplementary Figure 1. Stable HT-29 cells with thelenti-CRISPR/Cas9-NINJ2-KO constructwere further infected with lentivirus encoding *NINJ2 cDNA* construct ("NINJ2-OE") or empty vector, cultured in the selection medium with puromycin to achieve stable cell lines, "NINJ2-OE-Line-1/-2" and "Vec". Cells were cultured for the indicated time periods; *NINJ2 mRNA* and protein expression was tested by qPCR (**A**) and Western blotting (**B**) assays; Cell proliferation and apoptosis were tested by BrdU incorporation (**C**) and TUNEL staining (**D**) assays, respectively. "sg-C" stands for control cells with the non-sense control sgRNA. NINJ1 and NINJ2proteinexpressionwas normalized to the loading control Tubulin (B).For each assay, n=5.$^{\#}P$< 0.05 *vs.*"Vec" cells (no rescue). Experiments in this figure were repeated three times, and similar results were obtained.
